# Brain ventricular dimensions and relationship to outcome in adult patients with bacterial meningitis

**DOI:** 10.1186/s12879-015-1097-3

**Published:** 2015-08-25

**Authors:** Janni L. Sporrborn, Gertrud B. Knudsen, Mette Sølling, Karina Seierøe, Annette Farre, Bjarne Ø. Lindhardt, Thomas Benfield, Christian T. Brandt

**Affiliations:** Department of Pulmonary and Infectious Diseases, Hillerød Hospital, University of Copenhagen, Copenhagen, Denmark; Department of Infectious Diseases, Hvidovre Hospital, University of Copenhagen, Copenhagen, Denmark; Department of Diagnostic Radiology, Hvidovre Hospital, University of Copenhagen, Copenhagen, Denmark; Department of Clinical Biochemistry, Hvidovre Hospital, University of Copenhagen, Copenhagen, Denmark

## Abstract

**Background:**

Experimental studies suggest that changes in brain ventricle size are key events in bacterial meningitis. This study investigated the relationship between ventricle size, clinical condition and risk of poor outcome in patients with bacterial meningitis.

**Methods:**

Adult patients diagnosed with bacterial meningitis admitted to two departments of infectious diseases from 2003 through 2010 were identified. Clinical and biochemical data as well as cerebral computed tomographic images were collected. The size of the brain ventricles were presented as a Ventricle to Brain Ratio (VBR). Normal range of VBR was defined from an age matched control group. A multivariate analysis was performed to identify predictors of 30-day mortality.

**Results:**

One hundred and seven patients were included. Eighty-one patients had a CT scan at the time of diagnosis. VBR was identified as an independent risk factor of 30-day mortality, Mortality Rate Ratio: 6.03 (95 % confidence interval: 1.61-22.64, *p* = 0.008) for highest versus lowest tertile. A VBR deviating more than 2 standard deviations from the normal range was associated with increased mortality.

**Conclusions:**

Brain ventricles are commonly subject to marked changes in size as a consequence of meningitis. Increased brain ventricle size in the acute phase of bacterial meningitis was associated with increased mortality.

**Electronic supplementary material:**

The online version of this article (doi:10.1186/s12879-015-1097-3) contains supplementary material, which is available to authorized users.

## Background

Brain edema and hydrocephalus are severe clinical conditions leading to impaired consciousness and reduced brain function in patients suffering from bacterial meningitis.

Experimentally, changes in ventricular size have been shown to be closely related to disease severity and appear to be a significant event in meningitis pathophysiology [1, 2]. Among adult patients with bacterial meningitis, severe hydrocephalus is a clinical endpoint with high risk of poor outcome [3, 4].

In meningitis, the loss of blood-brain-barrier integrity leads to extracellular fluid accumulation and development of a vasogenic brain edema [5–7]. Compromised cerebral blood flow (CBF) autoregulation is observed in the early stages of meningitis, resulting in a vulnerable CBF dependent on the systemic blood pressure [8–11]. The phenomenon “Resistance to cerebrospinal fluid (CSF) outflow” indicates decreased re-absorption of CSF and therefore accumulation. This may, in extreme cases, cause development of hydrocephalus with accompanying increase of intracranial pressure (ICP) [12, 13].

As stated by the Kellie Monroe doctrine, increased volume in brain tissue, CSF or vascular compartment will result in an increased ICP and subsequently a decreased volume in the other compartments. This equation becomes apparent in the hydrocephalic brain where the ventricular system is enlarged on behalf of brain tissue. Similarly, in advanced stages of cerebral edema, a reduction or compression of the CSF spaces may be observed [14].

Hydrocephalus is diagnosed when extreme expansion of the ventricles is observed and is often defined by Evans ratio or the bi-caudate index (BCI) [15]. However, any ventricle expansion challenging the adaptive capacity of the brain and vascular compartment could have clinical implications and we believe that less pronounced changes in ventricular size, not diagnosed as hydrocephalus by radiological definitions, could be an important part of meningitis pathophysiology. The present study aimed to investigate the number of patients with bacterial meningitis presenting with ventricle compression or expansion and the association between ventricular dimensions and outcome.

## Methods

The study was conducted by retrospective data collection of patients who were admitted or referred to two departments of infectious diseases in the capital region from 2003 through 2010.

Patients were identified through the initial CSF sample with elevated leukocyte count in the biochemical databases LABKA I and LABKA II (*CSC Scandihealth Aarhus, Denmark*). Data on blood biochemistry were also acquired from LABKA I and II. Clinical data was collected from patient files, clinical data sheets, nurse registration files and discharge records. Data on computer tomographic (CT) images of the brain were collected from the radiological databases WebPAX (*Heart Imaging Technologies, Durham, North Carolina, USA*) and Web1000 (*Agfa, Mortsel, Belgium).*

### Inclusion criteria

Patients ≥16 years, admitted or referred to two Infectious Diseases specialist departments with fever and clinical symptoms of meningitis with headache, neck stiffness or reduced consciousness, coma or seizures. The clinical diagnosis of bacterial meningitis was supported by one or more of the following criteria:

1. CSF leukocyte >10 × 10^6^ cell/l in combination with a positive CSF culture or microscopy
2. CSF leukocyte >10 × 10^6^ cells/L and positive blood culture
3. CSF leukocyte count >100 × 10^6^ cells/L and CSF protein >2.2 g/L or CRP >80 mg/L.
4. CSF leukocyte count >100 × 10^6^ cells/L in combination with an otogenic focus.

Patients should have received an appropriate treatment regimen (dosage of antibiotics and treatment period).

### Exclusion criteria

Patients with age < 16 years or a primary admission to a paediatric department. Patients diagnosed with viral meningitis and encephalitis (either by PCR, intrathecal antibody synthesis or clinically), fungal or parasitic infection, cerebral abscess, nosocomial and post-surgical meningitis, primary endocarditis, aseptic meningitis, cerebral autoimmune disease or neoplasia were excluded.

Patients identified with confirmed or suspected bacterial meningitis according to these criteria should have had one or more CT scans performed during the course of disease to be included.

### Clinical data

Patient data were collected independently by two medical doctors to limit registration errors. Random cases were reviewed by a third medical doctor to verify data. In cases of disagreement files were reviewed again.

For each patient a datasheet was completed containing: 1) demographic information, 2) clinical values at the time of lumbar puncture (LBP) including blood pressure, pulse and temperature, 3) clinical status at presentation, 4) patient co-morbidity (based on discharge records and diagnosis from previous admissions), 5) results from CSF and blood biochemistry, 6) information on treatment (obtained from patient files, prescription sheets or the Danish Medicine Administration) and finally 7) patient outcome.

### Clinical presentation

As a Glasgow Coma Score (GCS) was not available for all included patients, level of consciousness on admission was determined from patient files and expressed as a Clinical Presentation Score (CPS): 1) comatose, 2) reduced consciousness, agitation, lack of ability to communicate, 3) drowsy, but able to communicate and 4) awake and alert.

### Outcome

Outcome was assessed retrospectively and established by available patient data from discharge or from the first ambulatory visit 10 to 14 days after discharge. Outcome was expressed according to the Glasgow Outcome Scale (GOS) [16]: 1) death, 2) vegetative state, 3) severe disability, 4) moderate disability and 5) good recovery .

### CT-imaging

All CT images were performed with patients in the supine position, untilted, using the anatomical landmarks of the scull base and vertex. Number and timing of CT scanning sessions were recorded for all included patients. Patients were divided into age groups (<30, 30–39, 40–49, 50–59, 60–69, 70–79 and ≥80) based on previously defined age-related changes of brain parenchyma in a normal population [17, 18]. Patients were also divided by time of scanning into: 1) patients who had a CT scan performed within 24 h of diagnosis and 2) patients who had a CT scan performed later in the course of disease.

### Measurement of ventricle size

From each collected CT scan, two representative and consecutive images, displaying the lateral ventricles at the level of the thalamus and upwards, where chosen. This was done to reduce the influence of variations in placing of slices and slice thickness around foramen of Monroe.

In imaging sessions with reduced slice thickness, three images were selected. Using MIPAV (http://mipav.cit.nih.gov/), a region of interest (ROI) was drawn lining ventricles and closely adjacent to brain cortex/cerebellum in each image calculating areas in mm^2^ and thereafter a Ventricle to Brain Ratio (VBR, see Additional file 1). The mean of the ratios from the included images constituted the patients VBR. VBR was measured by a person blinded to the clinical status and outcome of the patient.

### Control group

A control group of 115 individuals who had a brain CT scan diagnose ‘normal brain’ was included. The control group was recruited from the same population as the included meningitis patients and consisted of patients who had a CT scan performed due to e.g. headache, dizziness or unexplained syncope, in order to rule out focal brain pathology. Controls were randomly selected by the responsible radiologist (KS). Control group patients were evenly distributed in the age intervals:16–19, 20–29, 30–39, 40–49, 50–59, 60–69, 70–79, 80–89 and >90 years (*n* = 13 in each group except 20–29 years and 80–89 years where *n* = 12). As for meningitis patients, age groups 16–19 and 20–29 (<30) as well as age 80–89 and >90 (>80) were pooled for the final data analysis. Eight of the included controls had a history of alcohol abuse. VBR was calculated as described above and the results were implemented as our reference for normal range of VBR.

VBR values two standard deviations (2SD) above or below control group mean for each age interval was considered “*pathological*”. VBR values in-between +/− 2 SD were considered to be within normal range.

### Data analysis and statistics

Categorical variables were compared using *χ*^2^ statistics. Differences in continuous values were tested with the Mann-Whitney test. Mortality rate ratios (MRRs) with 95 % confidence intervals (CIs) were computed using Cox proportional hazard regression analysis. Patients with a CT scan performed within 24 h of diagnosis were included in a univariate analysis to identify risk factors of 30-day mortality. Variables that were associated with mortality in the univariate analysis at a significance level of *p* < 0.1 were included in a multivariate analysis.

Additionally, outcome was compared between patients with normal range VBR and patients with a pathological VBR. The analysis was performed both among patients who had a CT scan performed within 24 h of diagnosis and among all included patients, regardless of time of scanning.

Survival curves were constructed by the method of Kaplan-Meier. Analyses were performed using SPSS version 20.0 (SPSS Inc., Chicago, IL, USA) and Graph Pad Prism version 5.0 (GraphPad Software Inc., La Jolla, CA, USA). The study was approved by the Danish Data Protection Agency and by the Danish Health and Medicines Authority with record no.3-3013-135/1/.

## Results

### Patient characteristics, Table 1

We identified 1211 unique patients ≥16 years of age with a CSF cell count >10 × 10^6^ cells/L. Of those, 155 patients met the inclusion criteria. Four patients had had neurosurgical or ear-nose-throat surgery and were excluded due to having post-surgical meningitis. One patient could not be evaluated due to missing patient files. Of 150 patients, 109 had one or more CT scans during the course of hospitalization. Two patients were excluded due to insufficient quality of CT images.

**Table 1:** Characteristics of 81 patients with acute bacterial meningitis and CT imaging within 24 h of diagnosis according to 30-day survival

	All	Survivors	Non-survivors	*P* value
	*N* = 81	*N* = 55 (68 %)	*N* = 26 (32 %)	
Age, years	64 (52–74)	62 (51–68)	73 (54–84)	0.006
Sex				0.059
Male, %	44 (54 %)	34 (62 %)	10 (38 %)	
Female, %	37 (46 %)	21 (38 %)	16 (62 %)	
Etiology				0.23
Streptococcus pneumoniae	43 (53 %)	28 (51 %)	15 (58 %)	
Streptococcus sp.	6 (7 %)	2 (4 %)	4 (15 %)	
Staphylococcus aureus	4 (5 %)	3 (5 %)	1 (4 %)	
Listeria monocytogenes	2 (3 %)	1 (2 %)	1 (4 %)	
Unknown	21 (26 %)	16 (29 %)	5 (19 %)	
Other	5 (6 %)	5 (9 %)	0 (0 %)	
Adjuvant steroid, %				0.08
No	28 (35 %)	15 (27 %)	13 (50 %)	
Yes	52 (65 %)	39 (71 %)	13 (50 %)	
Comorbidity				0.39
None	51 (63 %)	32 (58 %)	19 (73 %)	
Alcoholism	13 (16 %)	8 (15 %)	5 (19 %)	
Malignancy	5 (6 %)	5 (9 %)	0 (0 %)	
Diabetes mellitus	5 (6 %)	4 (7 %)	1 (4 %)	
Asplenia	4 (5 %)	4 (7 %)	0 (0 %)	
Other immunosupp	1 (1 %)	1 (2 %)	0 (0 %)	
Other	2 (3 %)	1 (2 %)	1 (4 %)	
Change in mental status, %	61 (82 %)	39 (71 %)	22 (85 %)	0.20
Focal neurologic deficit, %	17 (24 %)	15 (27 %)	2 (8 %)	0.07
Seizures, %	5 (7 %)	4 (7 %)	1 (4 %)	1.0
Temperature, ° C, *n* = 64	38.9 (37.9-39.5)	38.8 (37.7-39.4)	39.0 (38.0-39.5)	0.49
MAP, mmHg, *n* = 64	102 (88–110)	103 (92–112)	94 (77–109)	0.18
White Blood Cell Count, 10^9^/L, *n* = 64	17.1 (12.1-22.6)	17.9 (12.5-24.0)	15.7 (11.9-22.9)	0.29
Plasma C-reactive Protein, mg/L	227 (144–337)	238 (151–334)	192 (87–369)	0.51
CSF-leukocytes	2182 (523–6188)	1932 (488–5150)	2826 (561–7930)	0.43
CSF-protein, *n* = 55	4.9 (2.5-7.8)	3.3 (2.1-6.2)	6.3 (5.1-11.2)	0.001
CSF-glucose, *n* = 59	1.5 (0.1-2.9)	1.4 (0.1-3.3)	1.5 (0.1-2.4)	0.40
VBR	0.10 (0.07-0.14)	0.09 (0.07-0.13)	0.13 (0.10-0.15)	0.005
VBR				0.005
<0.08	27 (33 %)	24 (44 %)	3 (11 %)	
0.08-1.2	25 (31 %)	17 (31 %)	8 (31 %)	
>0.12	29 (36 %)	14 (25 %)	15 (58 %)	

*MAP* mean arterial pressure, *CSF* cerebrospinal fluid, *VBR* ventricle to brain ratio

Patient age distribution was as follows: <30 (*n* = 5), 30–39 (*n* = 4), 40–49 (*n* = 12), 50–59 (*n* = 21), 60–69 (*n* = 29), 70–79 (*n* = 26) and ≥80 (*n* = 10).

Eighty-one patients had a CT scan performed in immediate relation to the diagnostic LBP (median −2 h before LBP [−5 to 0 h]) and were included in the primary study analysis. Of the remaining 26 patients, one had the first CT scan performed 72 h before diagnosis and 25 patients had their first CT scan performed more than 24 h after diagnostic LBP (median 85 h after LBP [51 to 149.5]). Only one patient presented with an imminent risk of interventricular obstruction due to a thalamic haemorrhage. The patient did not survive.

In Table 1, characteristics are shown for patients with a CT scanning performed in immediate relation to admission (*n* = 81). Including all patients, (*n* = 107) the most common etiologic agent was *Streptococcus pneumoniae (*42.0 %). Predisposing risk factors for infection were present in 32.0 % of patients with alcoholism and diabetes being the most common. Thirty-day study mortality was 33.0 %.

### Etiology – culture positive and negative cases

Among patients where a CT scan was performed within 24 h of admission we found no differences in CSF biochemistry (leukocyte count, protein and glucose) comparing culture positive to culture negative cases. CSF leukocyte counts (10^6^ cells/L), median 2724 [595 to 6985] versus 1166 [463 to 3332], *p* = 0.2; CSF protein (g/L), median 5.4 [2.7 to 8.3] versus 3.2 [1.8 to 4.6], *p* = 0.09; and CSF glucose (mmol/L), median 0.5 [0.1 to 2.4] versus 1.7 [1.5 to 3.3], *p* = 0.08.

### Clinical presentation (CPS)

Among patients where a CT scan was performed within 24 h of admission, the CPS was significantly correlated to GOS (Spearman Rank, rho = 0.24, *p* = 0.04). Among all patients diagnosed with bacterial meningitis (*n* = 150) CPS was also significantly correlated to GOS (Spearman Rank, rho = 0.32, *p* = 0.0002).

Patients with CT imaging performed within 24 h of admission had significantly lower CPS compared to patients where a CT scan was never performed (*p* = 0.004).

These patients were also more often treated in an intensive care ward (*n* = 44/81 versus *n* = 24/69, chi-square, *p* = 0.02) and more often received respirator treatment (*n* = 36/81 versus 19/69, *p* = 0.03).

### Ventricle to brain ratio – VBR, definition of pathological VBR, Fig. 1 and Table 2

**Fig. 1:**
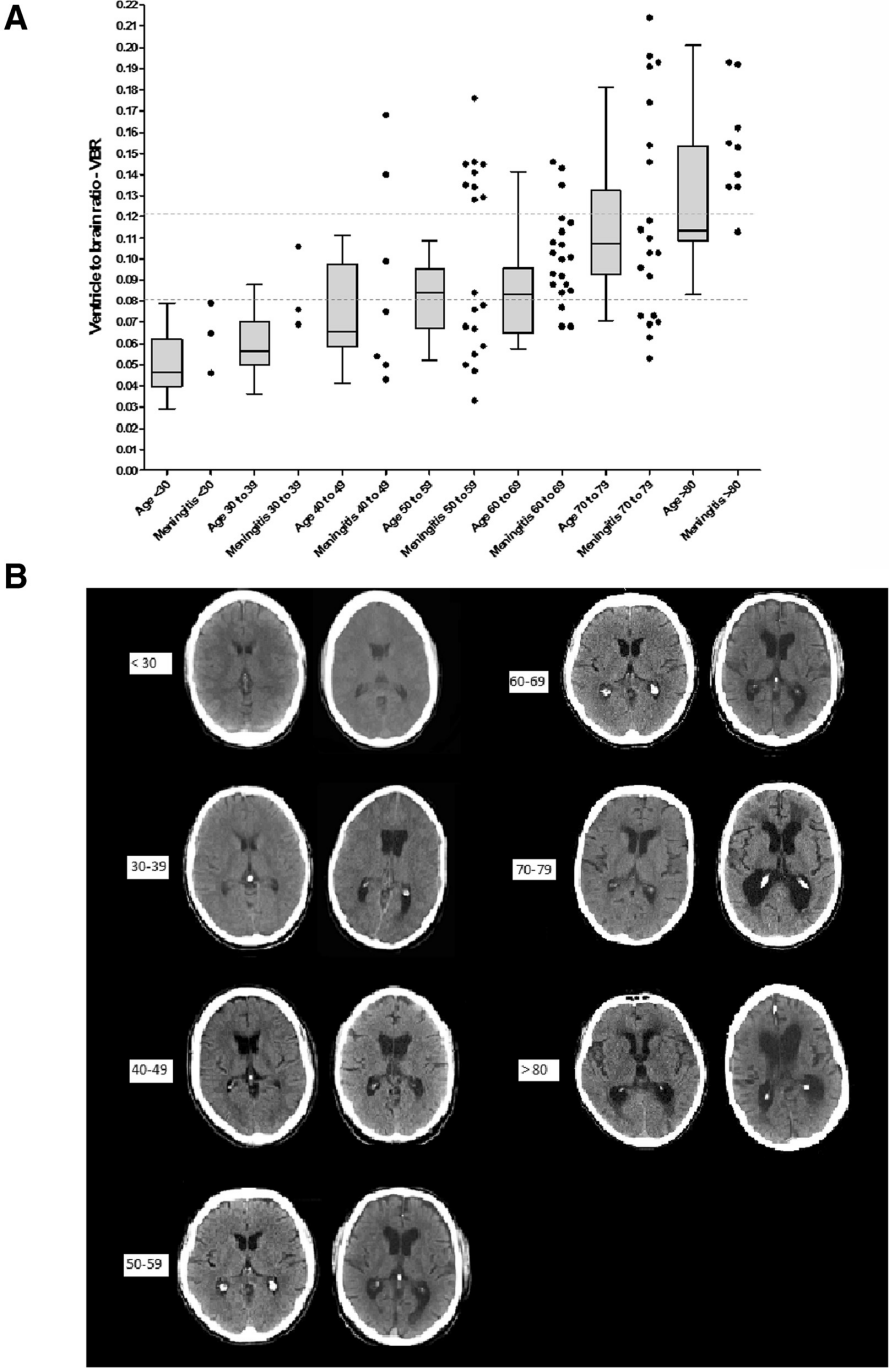
**a** Distribution of Ventricle to Brain Ratio in age groups. Patients (scatter plot) and controls (box and whiskers with box showing median and quartiles and whiskers min. and max.). Horizontal lines indicate VBR-tertiles used for multivariate analysis. **b** Examples of Ventricle to Brain Ratio in patients with bacterial meningitis. Normal range VBR (*left*) and VBR increased > 2SD (*right*) within each age interval

VBR increased with age and the variation in VBR was considerably greater in the age groups 70–79 years and > 80 years. Therefore the relationship between outcome and a pathological VBR was both performed including and excluding age groups >70 years (see Table 2 and Fig. 1a) [17].

**Table 2:** Pathological ventricle to brain ratio and outcome

CT scan performed within 24 h of diagnosis (*n* = 81)		Pathological VBR*	Normal VBR**	*P*-value
All age groups	No	28 % (*n* = 23/81)	72 % (*n* = 58/81)	
	GOS 1	56.5 % (*n* = 13/23)	31.0 % (*n* = 18/58)	0.033
	GOS 5	34.8 % (*n* = 8/23)	34.5 % (*n* = 20/58)	0.98
<70 years	No	33 % (*n* = 17/52)	67 % (*n* = 35/52)	
	GOS 1	53.0 % (*n* = 9/17)	14.3 % (*n* = 5/35)	0.003
	GOS 5	35.3 % (6/17)	42.9 % (*n* = 15/35)	0.66
All CT scans performed during admission (*n* = 107)				
All age groups	No	28.0 % (*n* = 30/107)	72.0 % (*n* = 77/107)	
	GOS 1	50.0 % (*n* = 15/30)	32.5 % (*n* = 25/77)	0.12
	GOS 5	26.7 % (*n* = 8/30)	37.7 % (*n* = 29/77)	0.37
<70 years	No	29.6 % (*n* = 21/71)	70.4 % (*n* = 50/71)	
	GOS 1	52.4 % (*n* = 11/21)	16.0 % (*n* = 8/50)	0.003
	GOS 5	28.6 % (*n* = 6/21)	48.0 % (*n* = 24/50)	0.19

*VBR* ventricle to Brain Ratio, *GOS* Glasgow outcome scale

* −/+ 2SD from control group mean VBR for age

** Within 2SD of control group mean VBR for age

Values of VBR denoted “pathological”, respectively 2 SD above and below control group mean in each age interval was: age 16–29 years (0.023 and 0.07), 30–39 (0.028 and 0.091), 40–49 (0.022 and 0.139), 50–59 (0.046 and 0.12), 60–69 (0.039 and 0.13), 70–79 (0.049 and 0.18) and >80 (0.071 and 0.19).

Among patients with a CT scan performed within 24 h of admission (*n* = 81), 28.4 % (*n* = 23/81) presented with a pathological VBR. Among patients with a first CT scan performed more than 24 h after diagnosis (*n* = 26), 27.0 % (*n* = 7/26) had a pathological VBR. Expansion of brain ventricles was observed in 25.0 % (*n* = 27/107) including all patients who had a CT scan performed.

Patients presenting with a pathological VBR had a significantly higher incidence of fatal outcome (GOS 1) compared to patients with a VBR within normal range (Table 2).

### Multivariate analysis of factors associated with 30-day mortality, Table 3 and Fig. 2

**Fig. 2:**
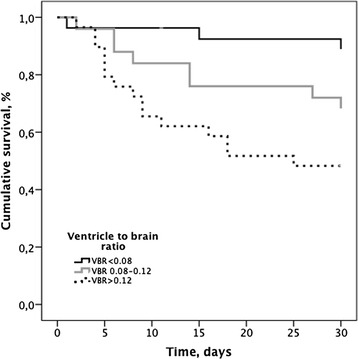
Survival from bacterial meningitis according to Ventricle to Brain Ratio. Log-rank test: *P* = 0.004

Eighty-one patients with a CT scan performed in association with the diagnostic LBP were included in the univariate analysis. Thirty-day mortality was 32.0 % (*n* = 26/81). Age, sex, corticosteroid treatment, CSF protein concentration and VBR were associated with 30-day outcome at a significance level of *p* < 0.1. There was no significant difference in remaining parameters, see Table 1. The relationship between VBR and survival is shown in Fig. 2.

**Table 3:** Multivariate analysis of predictors of 30-day mortality from acute bacterial meningitis

		Multivariate model 1		Multivariate model 2	
	Unadjusted MRR (95 % CI)	Adjusted MRR (95 % CI)	P-value	Adjusted MRR (95 % CI)	*P*- value
Age, per year increment	1.05 (1.01-1.08)	1.03 (0.99-1.06)	0.17	0.99 (0.95-1.04)	0.66
Sex					
Male	1.0	1.0		1.0	
Female	2.31 (1.05-5.10)	2.30 (0.99-5.29)	0.051	2.77 (0.96-8.00)	0.061
VBR			0.036		0.029
<0.08	1.0	1.0		1.0	
0.08-0.12	3.12 (0.83-11.75)	2.81 (0.70-11.28)	0.15	5.18 (0.88-30.53)	0.07
>0.12	5.97 (1.72-20.65)	6.03 (1.61-22.64)	0.008	8.78 (1.74-44.36)	0.009
Glucocorticosteroid					
No	1.0	1.0		1.0	
Yes	0.47 (0.22-1.01)	0.44 (0.19-1.03)	0.057	0.62 (0.20-1.96)	0.42
CSF-protein per g/L increment, *n* = 55	1.17 (1.07-1.28)	-		1.17 (1.04-1.31)	0.008

*MRR* mortality rate ratio, *CI* confidence interval, *VBR* ventricle to brain ratio, *CSF* cerebrospinal fluid

Because CSF-protein was unavailable in 26 of 81 patients, two multivariate models were performed. In model 1, age, sex, adjuvant steroid and VBR were included. After adjustment only VBR remained statistically significantly associated with 30-day outcome although both sex and steroid treatment approached significance (Table 3). In model 2, further adjustment was done for CSF protein. Here increasing VBR and higher levels of CSF protein were each associated with an increased risk of 30-day mortality (Table 3). Patients in the highest VBR tertile (>0.12) had a more than eight time increase in mortality when compared to patients with a VBR in the lowest tertile (<0.08) (MRR 8.78 (1.74-44.36), p = 0.009). Risk of 30-day mortality increased 17 % per 1 g/L increment of CSF protein. Adjuvant steroid treatment was not associated with outcome in this model.

## Discussion

We found increased mortality among patients who presented with significantly enlarged or compressed brain ventricles when compared to patients with a VBR within normal range. The association between increasing VBR and increased mortality was significant in a multivariate regression analysis, thereby taking into account both the increased mortality and variation in ventricle size observed with increasing age. In agreement with previous studies, the multivariate analysis also identified an augmented CSF protein content as a mortality risk factor in bacterial meningitis [19]. The level of CSF protein is a marker of the degree of blood-brain-barrier breakdown and may therefore be associated to both augmented vasogenic brain edema and to reduced CSF circulation and re-absorption caused by increased CSF viscosity [12, 20]. This could ultimately be associated to an increased risk of developing hydrocephalus [13].

The definition and classification of ‘hydrocephalus’ have for many years been a subject of debate [15, 21] Traditionally, hydrocephalus is diagnosed by use of single-point linear measurements such as the Bi-caudate Index (BCI) [22] or the Evans Ratio [23] although the reliability of these methods have been questioned [24]. We did not observe any cases of inter-ventricular obstruction and expansion was therefore believed to involve all parts of the ventricular system. As previously documented, a VBR determined from consecutive slices would provide a more accurate measure of ventricular changes than BCI or Evans ratio [25].

Our demonstration of plateau phases of ventricular space in age intervals - relatively constant up to the age of 60 and markedly increased in older individuals - is in accordance with previous findings [17]. Increasing ventricular size is a common finding with advancing age and the increased variance observed is likely due to accumulated brain tissue loss caused by age, infarctions, dementia, alcohol abuse and maybe psychiatric disease [26–28]. Due to variation, our definition of a *pathological* VBR was conservatively based on standard deviations calculated from the included control group, merely confirming the impact of VBR on outcome. The wide normal distribution increased the risk of regarding patients, in fact suffering from a state of hydrocephalus, and the predefined lower VBR limit is however likely to reflect only severe cortex and white matter edema. This hypothesis is supported by our finding of a stronger association between pathologically increased VBR and lethal outcome among patients below 70 years of age. The combination of both enlarged and compressed ventricles as *pathological* was performed since both entities are associated with ICP changes and clinical deterioration. Increased ICP is also what has recently been proposed by Glimåker *et al.* to be involved in poor outcome from bacterial meningitis. Repeated draining of CSF was their preferred method of reducing CSF pressure resulting in reduced mortality. We believe that our results support the findings by Glimåker *et al.*[29].

As would be expected, patients who had a CT scan performed within 24 h of admission had lower clinical presentation scores. Also, a higher proportion of these patients were treated in intensive care and received respirator treatment. Thus, they were likely to be sicker.

Even though we would expect the prognostic value of VBR to be evaluated in association with the clinical appearance of the patient, we did not detect any closer association in the outcome analysis. We believe that the reason may very well be that our retrospectively assessed clinical presentation score was too crude and that the patients, where a CT scan was performed within 24 h of admission, already presented very low scores.

Mortality in the present study was higher than in other recent studies [4, 30]. We believe this is to be expected since our patient population was considerably older and had much higher incidence of immunosuppressive co-morbidity (i.e. alcoholism, diabetes, and malignancy) compared to previous studies [31]. Both age and co-morbidity have previously been shown to be poor prognostic factors [31]. Furthermore, our patients with culture-confirmed bacterial meningitis represented pathogens with the most severe outcomes (streptococci and staphylococci) whereas we had no cases of meningitis caused *by N. meningitidis* which otherwise represent an infection with a fairly good prognosis [16, 28, 29]. One of the hospitals included in our study is a referral unit for patients with meningitis. This is likely to have influenced the number of patients with a complicated CNS infection and the relatively high incidence of ventricle expansion. The true incidence of hydrocephalus is however difficult to estimate, since previous studies have used different radiological definitions of hydrocephalus, presumably diagnosing only severe hydrocephalus as a clinical or imaging endpoint. Only one other study [3] has, to our knowledge, measured ventricular size unanimously in all patients and they present results very similar to ours. These numbers are much higher compared to studies where ventricle size was only assessed in patients with symptoms of increased ICP [4, 11, 30].

Forty patients had more than one CT scan during the course of disease. Ideally, these patients could have acted as their own controls, but unfortunately, the uneven timing of the imaging sessions made interpreting the time related variations in VBR speculative. Furthermore, repeated CT imaging was performed in patients with clinical deterioration or lack of improvement and not among patient with good recovery.

The major limitation of our study is the retrospective collection of data. Important clinical and biochemical data from admission was therefore incomplete. As previously mentioned our Clinical Presentation Score was rather crude and GOS could only be retrospectively assessed. The retrospective design did not allow us to further evaluate the pathophysiology behind the phenomenon of ventricle expansion. Resistance to CSF out-flow could be involved, and since ventricle expansion seem to develop irrespective of bacterial pathogen, host physiology or the inflammatory process itself may be responsible. Measuring CSF opening pressure is not routine in Denmark and this otherwise very relevant parameter therefore not available.

Our inclusion criteria may be considered to be wider than other clinical studies. However, our baseline data and number of culture negative cases are very similar to previous studies. Furthermore, inclusion of patients from a population with high levels of immunosuppressive co-morbidity could result in a significant bias if only CSF leukocyte count was evaluated. We did instead include c-reactive protein, that has been shown to be very sensitive as a supplemental parameter in order to differentiate viral from bacterial meningitis [32].

## Conclusions

The results of the present study show that enlarged brain ventricles in patients with bacterial meningitis are associated with increased mortality. More subtle changes in ventricle size other than frank hydrocephalus seem to be of clinical importance. Thus, serial brain imaging with estimation of ventricle expansion may therefore be useful supporting the clinical staging and prognosis of patients suffering from this disease. Possible treatment strategies following radiological suspicion of ventricle expansion in combination with clinical deterioration are limited due to the lack of evidence for many of these strategies - being medical or surgical treatments directed at reducing ICP.

Further research is needed to investigate the pathophysiology behind increasing ventricular size in order to elucidate whether ventricle enlargement - or the disease mechanism allowing ventricles to expand - are critical steps in the course of disease in bacterial meningitis.

## Additional file

Additional file 1:
**Calculation of ventricle to brain ratio using program MIPAV.** (TIFF 127 kb)
