# Supplementary figures and images for: Brain ventricular dimensions and relationship to outcome in adult patients with bacterial meningitis
Source: BMC Infect Dis. 2015 Aug 25;15:367. doi: 10.1186/s12879-015-1097-3 (PMC4547431; doi:10.1186/s12879-015-1097-3)

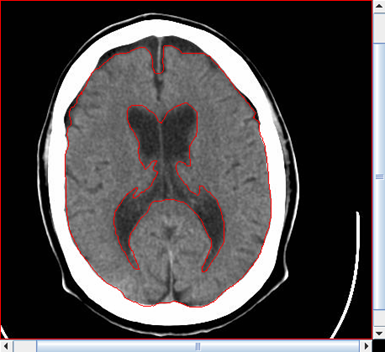

Supplement: Additional file 1: — Calculation of ventricle to brain ratio using program MIPAV. (TIFF 127 kb) [file 12879_2015_1097_MOESM1_ESM.tiff]
